# A Localization Method for Multistatic SAR Based on Convex Optimization

**DOI:** 10.1371/journal.pone.0142470

**Published:** 2015-11-13

**Authors:** Xuqi Zhong, Junjie Wu, Jianyu Yang, Zhichao Sun, Yuling Huang, Zhongyu Li

**Affiliations:** School of Electronic Engineering, University of Electronic Science and Technology of China, No.2006, Xiyuan Ave., West High-tech Zone, Chengdu 611731, P. R. China; Nankai University, CHINA

## Abstract

In traditional localization methods for Synthetic Aperture Radar (SAR), the bistatic range sum (BRS) estimation and Doppler centroid estimation (DCE) are needed for the calculation of target localization. However, the DCE error greatly influences the localization accuracy. In this paper, a localization method for multistatic SAR based on convex optimization without DCE is investigated and the influence of BRS estimation error on localization accuracy is analysed. Firstly, by using the information of each transmitter and receiver (T/R) pair and the target in SAR image, the model functions of T/R pairs are constructed. Each model function’s maximum is on the circumference of the ellipse which is the iso-range for its model function’s T/R pair. Secondly, the target function whose maximum is located at the position of the target is obtained by adding all model functions. Thirdly, the target function is optimized based on gradient descent method to obtain the position of the target. During the iteration process, principal component analysis is implemented to guarantee the accuracy of the method and improve the computational efficiency. The proposed method only utilizes BRSs of a target in several focused images from multistatic SAR. Therefore, compared with traditional localization methods for SAR, the proposed method greatly improves the localization accuracy. The effectivity of the localization approach is validated by simulation experiment.

## Introduction

Target localization is of significant importance for both monostatic and multistatic SAR applications. However, current studies on multistatic SAR mainly focus on imaging algorithm [1], synchronization [2] and experiments [3]. Very limited researches are reported on the target localization for multistatic SAR. Different from monostatic SAR, multistatic SAR has more complex geometrical relationship, as the transmitters and receivers are independent with each other. The double hyperbolic range equation leads to a double square root in the range equation of every T/R pair in multistatic SAR. So we can see that the localization of multistatic SAR, compared with monostatic SAR, is much more complicate. A range-Doppler (RD) approach for spaceborne monostatic SAR in [4] and airborne monostatic SAR in [5] are proposed. However, because the geometry configuration is much different between monostatic SAR and multistatic SAR, this method can only be used to monostatic SAR, but cannot be applied to multistatic SAR.

When it comes to multistatic SAR, in [6] a three-dimensional surface reconstruction method is proposed. It use the information of BRSs and phases of the target in each T/R pair image to solve the height of target. But it can not acquire the position information of X coordinate and Y coordinate of the target. In addition, the function of height is approximated to its first order Taylor formula. So when the height range become larger, its accuracy can not satisfy the request of localization.

On the other hand, The localization problem of multistatic SAR is similar to that of the multistatic radar system. There are some localization methods of multistatic radar system. However, localization accuracy in those algorithms can not satisfy the accuracy requirement for multistatic SAR images whose resolution is approximately 1m. In [7], Taylor-series estimation method is proposed. However, it can not capture a close enough starting point and has large computational burden. A new approach using LS algorithm is presented in [8] and [9]. The localization error is more than 10m, which is much larger than the resolution of the multistatic SAR images. So it can not be used in multistatic SAR localization. In addition, the localization precision of LS algorithm is much more sensible to BRS estimation error. It will be shown in the simulation experiment in this paper.

This paper describes a localization method for multistatic SAR, which can locate targets without DCE. The BRS is the only estimated parameter. So the accuracy of BRS estimation is most significant step, which will seriously influence the localization accuracy. The influence factor of BRS estimation error can be classified into two parts: 1) Time of arrival (TOA) estimation error. 2) Time synchronization error. In multistatic radar system, TOA estimation is a considerable challenge especially when the signal-to-noise ratio (SNR) is low. The multistatic SAR has same problem, which may affect the BRS estimation accuracy. In [10] and [11], TOA estimation algorithms are proposed to improve the estimation accuracy of TOA. As another main influence factor, time synchronization is also a big challenge for application of multistatic radar system as well as multistatic SAR. Without time synchronization, BRS cannot be estimated accurately. In [12], a time synchronization technology is presented for multistatic radar. [2] propose time synchronization technology for distributed SAR. Both of [12] and [2] can observably reduce the time synchronization between transmitters and receivers. So according to [12] and [2], we considerate the maximum of time synchronization error is 15us in simulation. In all, the BRS estimation error in multistatic SAR is less than 5m.

This method can be arranged into five steps: 1) Estimate the BRS of targets in each BSAR image. 2) Use those BRSs and position of transmitters and receivers build solving equations. 3) Construct target function, whose maximum position is target’s position. 4) Design a kind of iteration method to get target position. 5) Optimize the iteration process to greatly improve the accuracy and efficiency of the iteration method.

The fundamental localization principle is presented in Section II. PCA optimization method is described in Section III. Simulation results are shown in Section IV. In Section V, BRS estimation error simulation results are presented. The comparison between proposed method and LS algorithm in Section VI. At last, conclusion is shown in Section VII.

## Fundamental Localization Principle

**Fig 1:**
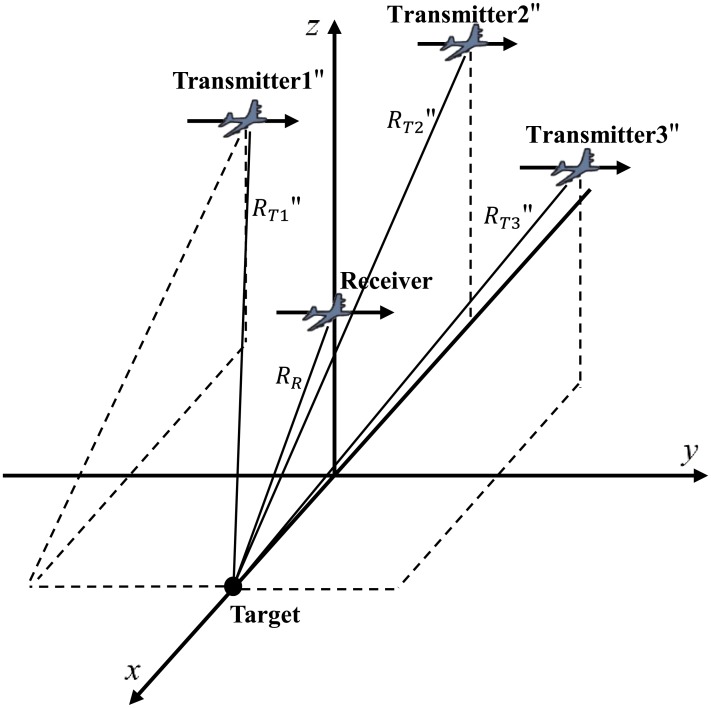
The Coordinate of Multistatic SAR System. Geometry coordinate system for the position relationship between transmitters, receiver and target.

The localization of a target in multistatic SAR is determined by the intersection of the iso-range lines, which are derived from the BRSs of each transmitter and receiver (T/R) pair. Different T/R pairs have different BRS characteristics. For a given range gate, the BRS is an ellipse in the imaging plane. The position of a target is the intersection of all ellipses of T/R pairs. To determine the localization of a point target in BSAR image, a common coordinate system must be established. Fig 1 illustrates the coordinate system adopted to present the position relationship between transmitters, receivers and target.

### Bistatic Range Sum Estimation

The slant range from the sensor to a target is defined by the equation

$$\begin{array}{c} \begin{array}{c} R=\sqrt{{\left(x_{T}-x\right)}^{2}+{\left(y_{T}-y\right)}^{2}+h_{T}^{2}}+\sqrt{{\left(x_{R}-x\right)}^{2}+{\left(y_{R}-y\right)}^{2}+h_{R}^{2}} \end{array} \end{array}$$ (1)

where (*x*, *y*), (*x*_*T*_, *y*_*T*_, *h*_*T*_) and (*x*_*R*_, *y*_*R*_, *h*_*R*_) are the localization of the target, transmitter and receiver respectively. The value of *R* at a particular time instant is determined by the effective delay of the pulse sampling window from the pulse transmission. As same as [4] and [5], we use time delay to estimate BRS. According to the range bin of target, the BRS is

$$\begin{array}{c} \begin{array}{c} R_{BRS}=c*\left(t_{offset}+\frac{index-\frac{N_{r}}{2}-1}{f_{s}}\right) \end{array} \end{array}$$ (2)

where c is the velocity of light, *t*_*offset*_ is the time delay of the range bin of $\frac{N_{r}}{2}+1$, *N*_*r*_ is the sample number in range, *f*_*s*_ is the sample frequency.

### Target Localization Theory

**Fig 2:**
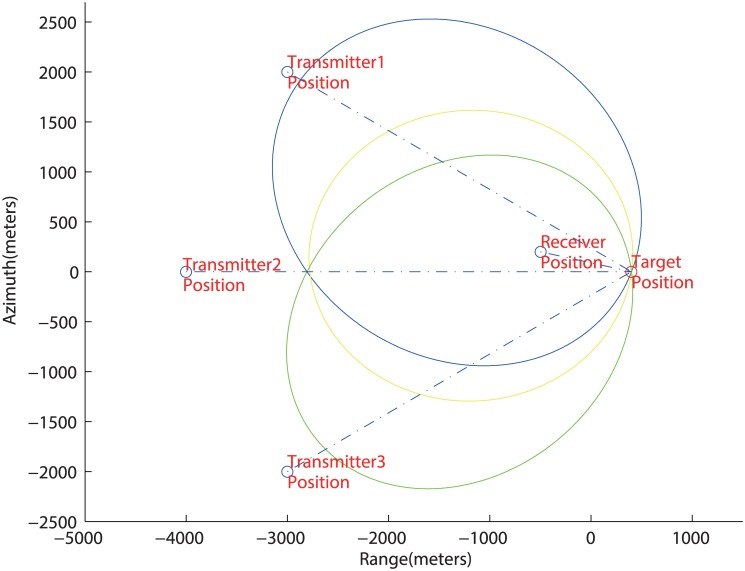
The Localization Principle of Multistatic SAR. Transmitters (red point), Receiver (red point) and BSAR Iso-range (ellipses).

After estimating the BRSs in every T/R pair, targets’ locations can be calculated by using Eq (1). Each BRS means an ellipse on the earth surface, as given in Fig 2.

The position of target is the intersection of those ellipses. So the target position is the only solution for BRS equations. Then a calculation method is presented for those equations in next subsection. For example, in Fig 1, the BRS equations are

R1=(xT1-x)2+(yT1-y)2+hT12+(xR-x)2+(yR-y)2+hR2R2=(xT2-x)2+(yT2-y)2+hT22+(xR-x)2+(yR-y)2+hR2R3=(xT3-x)2+(yT3-y)2+hT32+(xR-x)2+(yR-y)2+hR2 (3)

The position of target is the solution of Eq (3).

### Target Position Calculation Method

Because BRS equations Eq (3) are not only double hyperbolic but also overdetermined, the analytical solution cannot be found by mathematics method. So a method should be designed for solve the BRS equations. In the localization of multistatic SAR, we can see that from the information of only one T/R pair, the position of target can be located at every point of the ellipse which is derived by the BRS of the T/R pair. So inspired by convex optimization method, if there is a model function whose maximum is on the ellipse of a T/R pair, we can construct a target function through add all of model functions in each T/R pair. Then it means the position of the target is the maximum of the target function. At last, the position of the target can be obtained by convex optimization method.

#### Model Function

**Fig 3:**
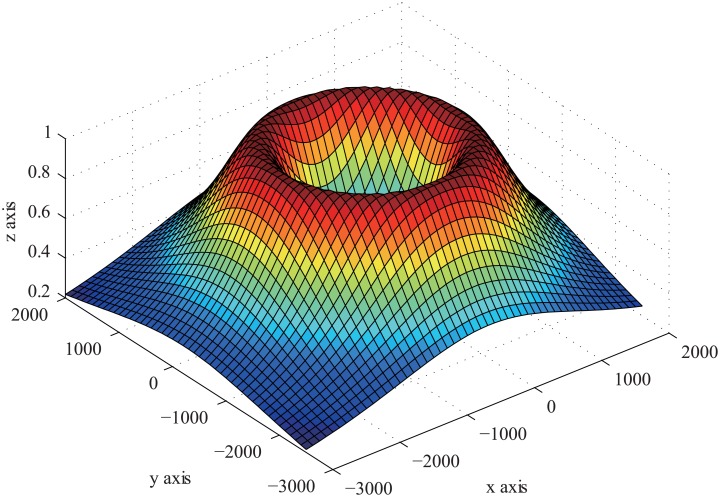
The Shape of Model Function.

For every T/R pair, the target is on the ellipse which is derived by the T/R pair. Model function is designed to

$$\begin{array}{c} \begin{array}{c} F\left(R\right)=\frac{1}{{\left[\ln(\frac{R^{2}}{R_{0}^{2}})\right]}^{2}+1} \end{array} \end{array}$$ (4)

where *R* is the BRS of a target on the earth surface, which is a fuction of coordinates x and y. *R*_0_ is the BRS of a target. In Fig 3, it shows that the model function can sufficiently present the target position information in each T/R pair.

#### Target Function

**Fig 4:**
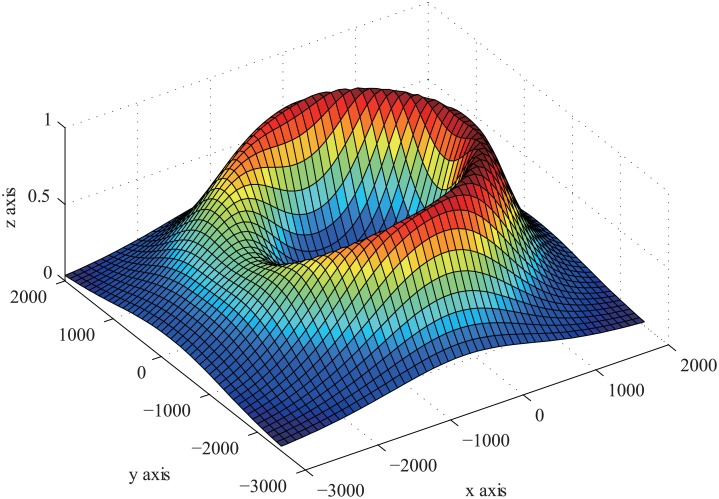
The Shape of Target Function.

**Fig 5:**
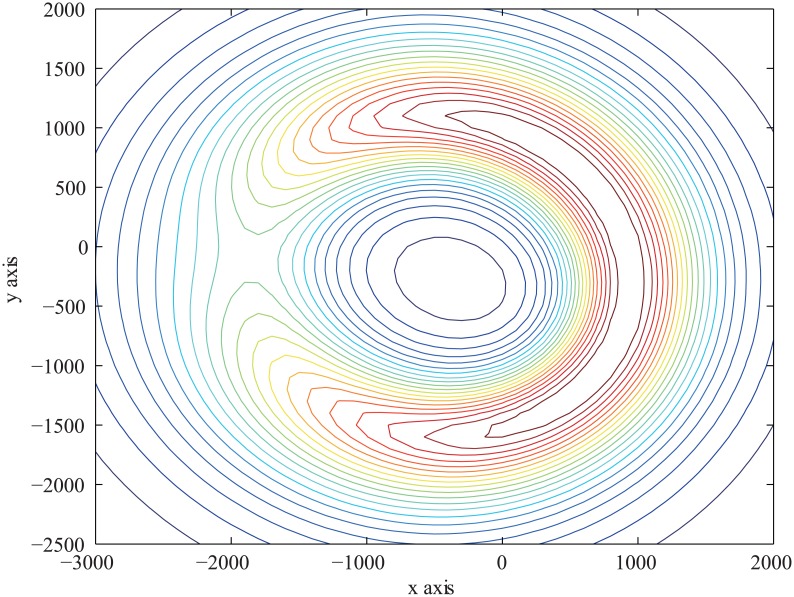
The Contour of Target Function.

Adding all model functions, we can get target function to be

$$\begin{array}{c} \begin{array}{c} F_{Target}=F_{1}+F_{2}+\cdots \end{array} \end{array}$$ (5)

where *F*_1_, *F*_2_ are model functions of different T/R pairs. The target function may be not a convex function, but the target function has only one maximum in domain of definition. So we can use convex optimization method to solve it. By Eq (3), the shape of the target function is shown in Fig 4. Its contour is shown in Fig 5.

### Gradient Descent Algorithm

After getting the target function, the gradients of the target function are derived repeatedly in every iteration step.

$$\begin{array}{c} \begin{array}{c} \begin{array}{c} GradVector_{1}=\left[\begin{array}{cc} \frac{\partial F_{1}}{\partial x} & \frac{\partial F_{1}}{\partial y} \end{array}\right]\\ GradVector_{2}=\left[\begin{array}{cc} \frac{\partial F_{2}}{\partial x} & \frac{\partial F_{2}}{\partial y} \end{array}\right]\\ \vdots \end{array} \end{array} \end{array}$$ (6)

where *F*_1*x*_ and *F*_1*y*_ are the partial derivatives of model function by x and y respectively. Then the iteration vector is the sum of normalized gradient vectors who multiply by Δ*R*_*i*_. So the new gradient vector is

$$\begin{array}{c} \begin{array}{cc} GradVector & =\frac{GradVector_{1}*\Delta R_{1}}{GradValue_{\max}}\\ & +\frac{GradVector_{2}*\Delta R_{2}}{GradValue_{\max}}\\ & +\cdots \end{array} \end{array}$$ (7)

where

$$\begin{array}{c} \begin{array}{c} \Delta R_{1}=R_{b1}-R_{1} \end{array} \end{array}$$ (8)

$$\begin{array}{c} \begin{array}{c} \Delta R_{2}=R_{b2}-R_{2} \end{array} \end{array}$$ (9)

$$\begin{array}{c} \begin{array}{c} GradValue_{\max}=\max\left\{GradValue_{1},GradValue_{2},\cdots \right\} \end{array} \end{array}$$ (10)

*R*_*b*1_ and *R*_*b*2_ are BRSs of iteration point in different BSAR combinations, *R*_1_ and *R*_2_ are the BRSs of targets in different BSAR combinations, *GradValue*_max_ is the normalization factor, *GradValue*_1_ and *GradValue*_2_ are 2-norm of *GradVector*_1_ and *GradVector*_2_ respectively. $\frac{GradVector_{i}*\Delta R_{i}}{GradValue_{\max}}$ is gradient vector for the *i*th T/R pair. *GradVector* is the sum of all T/R pair gradient vectors.

## PCA Optimization Method

**Fig 6:**
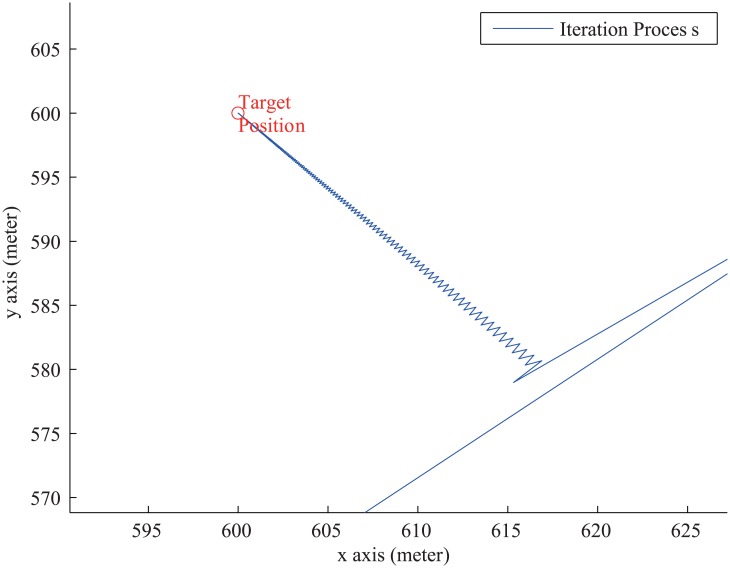
The Noise in Iteration Process.

After obtaining the gradient iteration vector by Eq (7), the position of the target can be calculated by iteration calculation. But if Eq (7) is used to locate the position of the target, we will find serious noises in the iteration process, which is shown in Fig 6.

Because when the iteration point approach to target position step by step, the shape of target function become much too sharp, which results in large value of the gradient vector within a small position deviation. The phenomenon is similar to simple harmonic motion in physics. Gradient vector represents the restoring force.

Due to the noise, the localization method faces two serious problems: 1) The noise results in heavy computational burden. Most of the iterations are wasted in noise. 2) Under the influence of noise, the accuracy of target position can’t be guaranteed obviously. In order to find the accurate position of the target, an optimization algorithm is designed to deal with the problem. The algorithm can effectively remove the iteration noises, which is presented in next subsection.

### Iteration Process Optimization

Principal component analysis (PCA) is a classic algorithm in pattern recognition. It is a kind of statistical procedure that uses an orthogonal transformation to convert a set of observations of possibly correlated variables into a set of values of linearly uncorrelated variables called principal components (the first principal component has the largest possible variance).

Back to our algorithm, when the iteration calculation result is closer to target position, the noise will become the largest possible variance component in gradient vector. The noise vector is always not only orthogonal with right gradient vector, but also the maximum component in gradient vector. Based on characteristic of PCA, which is described above, it is an effective method to find noise vector in gradient vectors. By filtering out the noise vector, it can greatly improve the efficiency of iteration algorithm.

#### The Mean-removed Gradient Vector

In order to find the noise vector, the mean-removed vector of gradient is firstly required. The matrix of gradient vector is

$$\begin{array}{c} \begin{array}{c} P=\left[\begin{array}{c} GradVector_{1}\\ GradVector_{2}\\ \begin{array}{c} \vdots \\ GradVector_{N} \end{array} \end{array}\right] \end{array} \end{array}$$ (11)

The mean vector of gradient is

$$\begin{array}{c} \begin{array}{c} GradVector_{mean}=\frac{1}{N}\left(GradVector_{1}+GradVector_{2}+\cdots +GradVector_{N}\right) \end{array} \end{array}$$ (12)

So the mean-removed gradient vector matrix is

$$\begin{array}{c} \begin{array}{c} \tilde{P}=\left[\begin{array}{c} GradVector_{1}-GradVector_{mean}\\ GradVector_{2}-GradVector_{mean}\\ \vdots \\ GradVector_{N}-GradVector_{mean} \end{array}\right] \end{array} \end{array}$$ (13)

#### Analysis Noise Based on PCA

After obtaining the mean-removed gradient vector matrix, we can use it to get the covariance matrix of gradient vector. It is given by

$$\begin{array}{c} \begin{array}{c} C={\tilde{P}}^{T}\tilde{P} \end{array} \end{array}$$ (14)

Doing eigenvalue decomposition with covariance matrix, we can get

$$\begin{array}{c} \begin{array}{c} C=U^{T}\Lambda U \end{array} \end{array}$$ (15)

where Λ is the diagonal matrix of eigenvalues *λ*_*k*_ of ${\tilde{P}}^{T}\tilde{P}$, *U* is eigenvectors of ${\tilde{P}}^{T}\tilde{P}$. The eigenvector of the maximum *λ*_*k*_ is the noise vector.

#### Filtering Noise Based on PCA

From PCA, we know the vectors and weights of the real gradient vector and the noise vector in gradient vector. So the noise can be weaken by the information from PCA. The weight of each component in gradient vector is given by

$$\begin{array}{c} \begin{array}{c} W=U*GradVector^{T} \end{array} \end{array}$$ (16)

So the final gradient vector is given by

$$\begin{array}{c} \begin{array}{c} GradVector_{final}=\Delta \bar{R}*{\left[U^{T}*\Lambda^{-1}*W\right]}^{T}/\lambda_{\max} \end{array} \end{array}$$ (17)

where $\Delta \bar{R}=\frac{1}{N}(\left|\Delta R_{1}|+|\Delta R_{2}|+\cdots +|\Delta R_{N}\right|)$, *λ*_max_ = max{*λ*_1_, *λ*_2_,…, *λ*_*N*_}. In the function, *W* is the projection of gradient vector in noise vector and real vector. Because Λ is the diagonal matrix of the weight of noise vector and real vector. So the Λ^−1^*W can weaken the noise component and strengthen the real component. From [*U* ^T^*Λ^−1^*W]^*T*^, the gradient vector is recalculated by using modified weigh Λ^−1^*W. The *λ*_max_ is normalization factor and the $\Delta \bar{R}$ is the mean step size of all T/R pairs.

In the end, when the iteration calculation result reaches the nearby position of the target position, there is only noise left. So we can use other attenuation factors to weaken the noise. In proposed method, the attenuation factor is

a=minΔR¯C0,1 (18)

where the value of *C*_0_ is determined by actual situation. If $\Delta \bar{R}<C_{0}$, we can let the final gradient vector

$$\begin{array}{c} \begin{array}{c} GradVector_{final}^{\prime }=GradVector_{final}*\left|\frac{\Delta \bar{R}}{C_{0}}\right| \end{array} \end{array}$$ (19)

**Fig 7:**
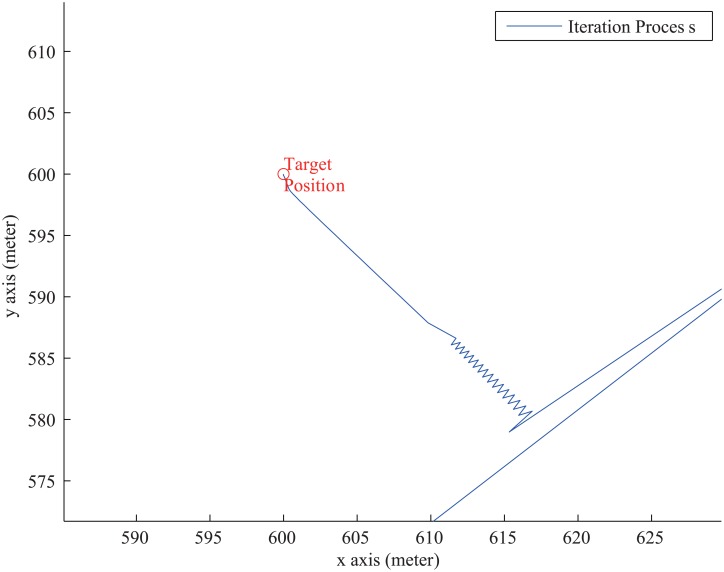
The Iteration Process After PCA Optimization.

The iteration process is shown in Fig 7. From Fig 7, we can see that after filtering noises by PCA, there are not noise in iteration process.

So the iteration equation is given by

$$\begin{array}{c} \begin{array}{c} P_{i+1}=P_{i}+GradVector_{final} \end{array} \end{array}$$ (20)

where *P*_*i*_ is current iteration point vector, *P*_*i*+1_ is next iteration point vector.

## Numerical Simulation

To verify the effectiveness of the proposed localization method based on convex optimization, we carry out numerical simulation in this section. The simulation parameters are listed in Table 1. Multistatic SAR is set to be one receiver and multiple transmitters. This configuration guarantees the imaged scenes in different T/R pairs to be the same, which is convenient to match targets between these focused images.

**Table 1:** Simulation Parameters.

Parameter	Value
Localization of Receiver	(0, 0, 0.4)km
Localization of First Transmitter	(−6, 4, 6)km
Localization of Second Transmitter	(−8, 0, 6)km
Localization of Third Transmitter	(−6, −4, 6)km
Velocity of Receiver	50ms
Velocity of First Transmitter	50ms
Velocity of Second Transmitter	50ms
Velocity of Third Transmitter	50ms
Center frequency	9.65 GHz
Range Bandwidth	200MHz
Chirp Sign Duration	5us
PRF	500Hz

**Fig 8:**
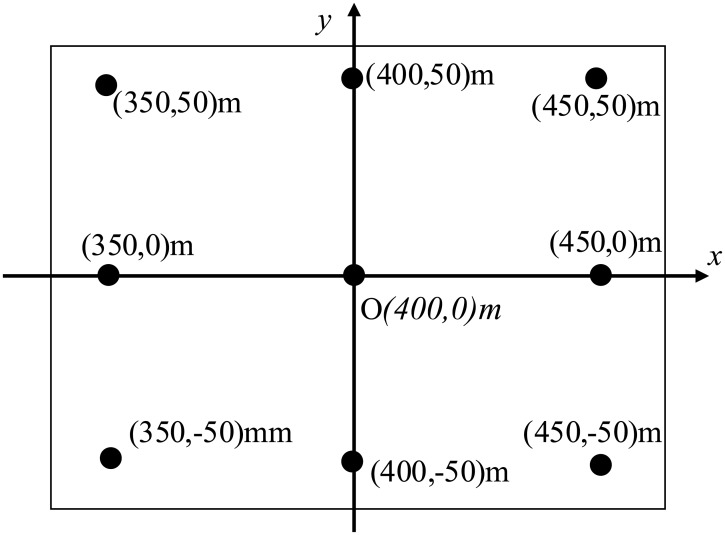
Target area used in the simulation.

In order to highlight the capacity of locating in the scene, we set 9 point targets, which forms a 3 × 3 matrix as shown in Fig 8. The distance between two adjacent targets along X axis direction and Y axis direction is 100m. Target *O* is set to be the reference target.

The imaging accuracy of multistatic SAR is also an important factor in localization method. As the only estimated parameter, the BRS plays a significant role in localization accuracy. In other word, if a target is focused in wrong bistatic sum range cell, the localization of the target can not be obtained in the proposed method. So in order to obtain accurate SAR images, NuSAR algorithm in [13] is presented, where transfer functions are calculated numerically. In NuSAR algorithm, the range cell migration of targets can be accurately calculated and imaging precision is guaranteed. So it is used in the proposed method to deal with images of BSARs.

**Fig 9:**
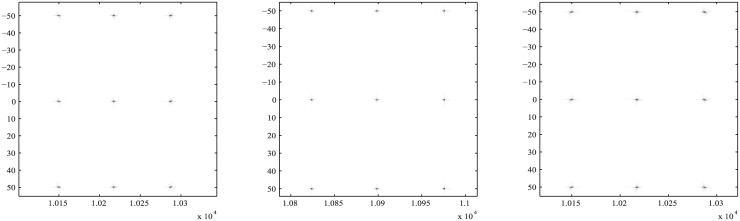
Focused Images. (a) The focused image of first T/R pair; (b) The focused image of second T/R pair; (c) The focused image of third T/R pair.

From Fig 9, we can get the range and azimuth index of the nine targets in the three T/R pairs of multistatic SAR. The localization results are shown in Table 2. Because the BRS in multistatic SAR image is discrete, the BRS can not be accurately obtained from those images, which lead to localization error in localization results.

**Table 2:** Simulation results without BRS estimation error.

Target	True coordinates	Localization Result with PCA	Error with PCA	Localization Result without PCA	iterations with/without PCA
1	(350, −50)m	(349.8617, −49.9613)m	0.1436m	(349.8560, −49.9525)m	86/794
2	(400, −50)m	(400.0146, −49.9462)m	0.0557m	(400.0000, −49.9151)m	71/539
3	(450, −50)m	(450.2433, −49.9199)m	0.2561m	(450.2431, −49.9147)m	123/559
4	(350, 0)m	(349.8673, −0.0173)m	0.1339m	(349.8560, 0.0475)m	79/789
5	(400, 0)m	(399.9994, −0.0165)m	0.0165m	(400.0000, 0.0170)m	115/2530
6	(450, 0)m	(450.2471, 0.0645)m	0.2554m	(450.2431, 0.0851)m	103/555
7	(350, 50)m	(349.8496, 49.9716)m	0.1530m	(349.8560, 50.0475)m	89/781
8	(400, 50)m	(400.0176, 50.0256)m	0.0310m	(400.0000, 50.0849)m	91/526
9	(450, 50)m	(450.2277, 49.9640)m	0.2305m	(450.2431, 50.0852)m	101/546

## BRS Estimation Error Simulation

In the proposed method, the accuracy of BRS estimation is the most important influence factor of localization accuracy. The influence of BRS estimation error is shown as follows.

The BRS error experiments use the Table 1 configure of multistatic SAR, and the target area is as same as Fig 8. The localization result of 0.5m and 1m BRS estimation error is shown in Tables 3 and 4 respectively.

**Table 3:** 0.5m BRS Estimation Error Simulation Results.

Target	True coordinates	Localization Result with PCA	Error with PCA	Localization Result without PCA	iterations with/without PCA
1	(350, −50)m	(350.6059, −49.8785)m	0.6179m	(350.5867, −49.6989)m	66/95
2	(400, −50)m	(400.3737, −49.9013)m	0.3865m	(400.3588, −49.8508)m	66/322
3	(450, −50)m	(450.5893, −49.8571)m	0.6064m	(450.5911, −49.8499)m	113/334
4	(350, 0)m	(350.5986, 0.1325)m	0.6131m	(350.5918, 0.1774)m	66/99
5	(400, 0)m	(400.3383, −0.1186)m	0.3584m	(400.3588, 0.1494)m	71/317
6	(450, 0)m	(450.5965, 0.1140)m	0.6073m	(4450.5911, 0.1502)m	102/329
7	(350, 50)m	(350.5939, 50.0465)m	0.5957m	(350.5917, 50.1781)m	67/91
8	(400, 50)m	(400.3808, 50.0647)m	0.3863m	(400.3588, 50.1496)m	76/308
9	(450, 50)m	(450.5519, 49.9457)m	0.5545m	(450.5911, 50.1503)m	94/320

**Table 4:** 1m BRS Estimation Error Simulation Results.

Target	True coordinates	Localization Result with PCA	Error with PCA	Localization Result without PCA	iterations with/without PCA
1	(350, −50)m	(351.5086, −49.8378)m	1.5173m	(351.5051, −49.8054)m	90/224
2	(400, −50)m	(401.5729, −49.9483)m	1.5738m	(401.5725, −49.9193)m	103/310
3	(450, −50)m	(451.7748, −49.8969)m	1.7778m	(451.7726, −49.8839)m	117/293
4	(350, 0)m	(351.4644, −0.1215)m	1.4695m	(351.5050, 0.1939)m	67/220
5	(400, 0)m	(401.5638, −0.0451)m	1.5645m	(401.5725, 0.0810)m	96/305
6	(450, 0)m	(451.7731, 0.0960)m	1.7757m	(451.7726, 0.1162)m	108/288
7	(350, 50)m	(351.4525, 49.9599)m	1.4531m	(351.5050, 50.1940)m	75/212
8	(400, 50)m	(401.5920, 50.0446)m	1.5926m	(401.5725, 50.0805)m	79/297
9	(450, 50)m	(451.7553, 49.9515)m	1.7560m	(451.7726, 50.1165)m	98/279

**Fig 10:**
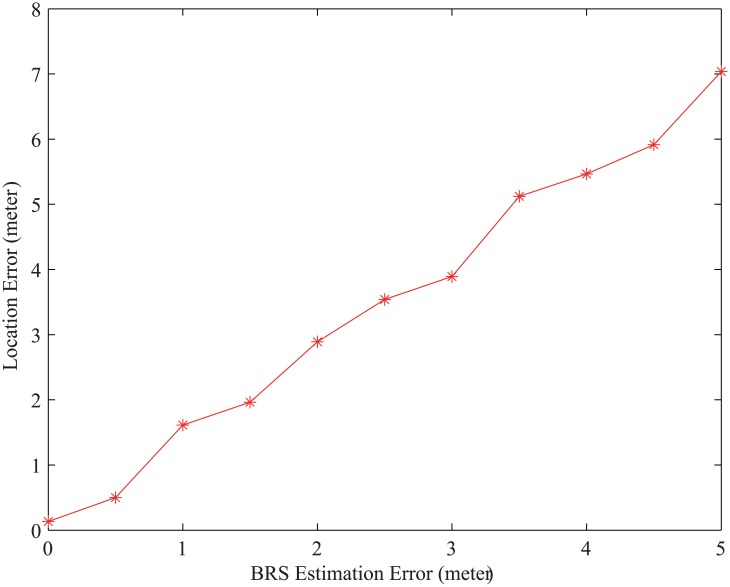
Localization Error under different BRS Estimation Errors.

The value of BRS estimation error varies from 0m to 5m, and the curve of localization error is shown in Fig 10. The relationship between BRS estimation error and localization error is proximate to linear relationship, and the slope of the linear is approximate to 1.4.

## Comparison between Proposed Method and LS Algorithm

In this section, the advantage of the proposed method will be investigated through comparing with LS algorithm. The simulation parameters are listed in Table 5. Because the LS algorithm in [8] and [9] is used in monostatic range case, but in multistatic SAR case it is BRS. In order to do the simulation, we set targets to be receivers only for LS algorithm.

**Table 5:** Simulation Parameters.

Parameter	Value
Localization of Receiver (Not for LS algorithm)	(0, 0, 0.4)km
Localization of First Transmitter	(−6, 4, 6)km
Localization of Second Transmitter	(−8, 0, 6)km
Localization of Third Transmitter	(−6, −4, 6)km
Target Position (Receiver for LS algorithm)	(0.4, 0, 0)km

Then the localization precision of the two method is shown in Table 6. In Table 6, we can see that the localization precision of LS algorithm is too much sensible for range estimation error to be used in multistatic SAR system. So the proposed method is a suitable localization method for multistatic SAR system.

**Table 6:** The comparison between proposed method and LS algorithm.

Target Position	Range Estimation Error	Proposed Method	LS aglorithm	Localization Error of Proposed Method/LS aglorithm
(400, 0, 0)m	(0.0000, 0.0000, 0.0000)m	(400.0000, 0.0017)m	(400.0000, 0.0000)m	0.0017m/0.0000m
(400, 0, 0)m	(0.1827, 0.1265, 0.0195)m	(400.0749, −0.0391)m	(400.1654, −0.1967)m	0.0845m/0.2570m
(400, 0, 0)m	(0.3719, −0.2739, 0.3765)m	(400.1956, 0.0055)m	(396.7823, 0.0055)m	0.1957m/3.2177m
(400, 0, 0)m	(0.5486, −0.0175, 0.3603)m	(400.2435, −0.2269)m	(397.7185, −0.2269)m	0.3328m/2.2928m
(400, 0, 0)m	(−0.5730, −0.1252, 0.6652)m	(399.9850, 1.4923)m	(399.1316, 1.4922)m	1.4924m/1.7265m
(400, 0, 0)m	(0.5844, 0.9190, 0.3115)m	(400.4396, −0.3289)m	(402.5839, −0.3290)m	0.5490m/2.6048m
(400, 0, 0)m	(−1.1143, 0.8379, 1.0416)m	(400.2043, 2.5978)m	(404.4999, 2.5982)m	2.6058m/5.1961m
(400, 0, 0)m	(−0.3018, 0.4353, −0.9207)m	(399.2999, −0.7451)m	(405.1934, −0.7459)m	1.0224m/5.2467m
(400, 0, 0)m	(−1.4523, −1.2892, 1.0351)m	(399.5587, 2.9982)m	(394.3516, 2.9976)m	3.0305m/6.3945m
(400, 0, 0)m	(0.7793, −0.7316, 1.8009)m	(400.3618, 1.2318)m	(390.0043, 1.2314)m	1.2838m/10.0713m
(400, 0, 0)m	(1.2745, 1.4170, −1.5030)m	(400.2121, −3.3457)m	(407.8644, −3.3474)m	3.3524m/8.5472m
